# Preconception care: promoting reproductive planning

**DOI:** 10.1186/1742-4755-11-S3-S2

**Published:** 2014-09-26

**Authors:** Sohni V Dean, Zohra S Lassi, Ayesha M Imam, Zulfiqar A Bhutta

**Affiliations:** 1 Division of Women and Child Health, Aga Khan University Karachi, Pakistan

**Keywords:** teen pregnancy, preconception, pregnancy intervals, birth spacing

## Abstract

**Introduction:**

Preconception care recognizes that many adolescent girls and young women will be thrust into motherhood without the knowledge, skills or support they need. Sixty million adolescents give birth each year worldwide, even though pregnancy in adolescence has mortality rates at least twice as high as pregnancy in women aged 20-29 years. Reproductive planning and contraceptive use can prevent unintended pregnancies, unsafe abortions and sexually-transmitted infections in adolescent girls and women. Smaller families also mean better nutrition and development opportunities, yet 222 million couples continue to lack access to modern contraception.

**Method:**

A systematic review and meta-analysis of the evidence was conducted to ascertain the possible impact of preconception care for adolescents, women and couples of reproductive age on MNCH outcomes. A comprehensive strategy was used to search electronic reference libraries, and both observational and clinical controlled trials were included. Cross-referencing and a separate search strategy for each preconception risk and intervention ensured wider study capture.

**Results:**

Comprehensive interventions can prevent first pregnancy in adolescence by 15% and repeat adolescent pregnancy by 37%. Such interventions should address underlying social and community factors, include sexual and reproductive health services, contraceptive provision; personal development programs and emphasizes completion of education. Appropriate birth spacing (18-24 months from birth to next pregnancy compared to short intervals <6 months) can significantly lower maternal mortality, preterm births, stillbirths, low birth weight and early neonatal deaths.

**Conclusion:**

Improving adolescent health and preventing adolescent pregnancy; and promotion of birth spacing through increasing correct and consistent use of effective contraception are fundamental to preconception care. Promoting reproductive planning on a wider scale is closely interlinked with the reliable provision of effective contraception, however, innovative strategies will need to be devised, or existing strategies such as community-based health workers and peer educators may be expanded, to encourage girls and women to plan their families.

## Introduction

For 1.8 billion adolescents who will be the next generation of adults, this is a pivotal time in their lives- of challenges and opportunities [1]. Preconception care recognizes that many adolescent girls and young women will be thrust into motherhood without the knowledge, skills or support they need; and that by promoting health and providing preventive care, we are investing in better outcomes for them and their children. For this review, we defined preconception care as “any intervention provided to women and couples of childbearing age, regardless of pregnancy status or desire, *before* pregnancy, to improve health outcomes for women, newborns and children” (detailed discussion of the importance and scope of preconception care is given elsewhere [2]). Using the lifecycle approach to provide a continuum of care [3] ensures that gains in childhood are built upon during adolescence, and that adolescent girls and boys are prepared for their transition to becoming adults, and potentially parents. Many adolescent girls and young women face challenges such as interpersonal violence, coerced intercourse, sexually-transmitted infections (STIs) especially HIV, under-nutrition or obesity and their health consequences which makes them highly vulnerable. Additionally, social pressures prevent them from completing their education and force them into early marriages and childbearing. In low- and middle-income countries (LMICs), 30% of girls are married before the age of 18; and worldwide approximately 16 million adolescent girls give birth, while 3 million pregnant adolescents undergo unsafe abortions [4].

Pregnancy in adolescence portends greater risk to the mother and newborn- including anemia, mortality, stillbirths, and prematurity- since adolescent girls are not yet physically mature themselves [5]. Adolescent girls are two to five times more likely to die from pregnancy-related causes than women age 20-29 years [4]. In many contexts, their situation is further complicated by a number of factors including poverty, lack of education, restricted access to care, weak health systems that are not sensitive to their needs, abuse, unplanned or unwanted pregnancies, and the absence of autonomy or support in their social arrangement. Adolescent girls who become pregnant are limited in their educational and employment opportunities, disadvantaging themselves and their children [6]. Conversely, adolescent girls completing secondary education are less likely to marry early or get pregnant, and those who do become pregnant are more likely to have well-nourished babies who survive the neonatal period [1].

Improving health and preventing pregnancy in adolescence should be consolidated through better reproductive health for young people. Young adulthood is an opportune time in the preconception period to encourage women and couples to consider developing a reproductive plan. Reproductive planning, the freedom for women (and couples) to choose when, how often and how many children they wish to have, has a direct impact on women’s health and their pregnancy outcomes. Reproductive planning is an important and fundamental component of preconception care, which could reduce adolescent pregnancy rates, and promotes spacing between pregnancies.

Reproductive planning has the potential to considerably reduce maternal, newborn, infant and child deaths. It could decrease 71% of unwanted pregnancies, thereby eliminating 22 million unplanned births, 25 million induced abortions and 7 million miscarriages [7]. Reproductive planning has other far-reaching effects. It could substantially avert sexual transmission of HIV through correct and consistent condom use. It would likely lead to smaller families, giving women more opportunities for paid employment and civic participation, and allowing parents to invest more in their children’s health, education and well-being. This would further slow population growth, reduce poverty and improve development, resulting in greater equity especially in the poorest regions with the highest burden of pregnancy-related death and disability [8,9].

Women and couples need access to safe, effective, acceptable and affordable methods of contraception to plan the timing, spacing and number of their children. This requires that they are well-informed of the correct and consistent use of these methods and the benefits of reproductive planning so that they are able to exercise choice and control over their own reproductive health. Counseling simultaneously ties in to reproductive planning and preconception care; so that pregnancies are more likely to be intended and appropriate healthcare services can be provided before pregnancy [10].

Millions of women and children, and future generations can benefit if we invest in preconception care (intervention targeted during pre-pregnancy and inter-pregnancy period) to improve adolescent health and encourage reproductive planning This paper presents the findings of a systematic review that was undertaken to consolidate the evidence for risks and interventions relating adolescent health and reproductive planning during preconception period. It begins with addressing the increased risks of pregnancy in adolescent girls and interventions to promote adolescent health and prevent teen pregnancy. The next section highlights the importance of birth spacing. An outline of pregnancy risks at advanced maternal age and genetic counseling follows. The final section illustrates how preconception counseling can be an effective means to encourage reproductive planning, reduce risk and enhance health before pregnancy.

## Methods

We systematically reviewed all literature published up to 2011 to identify studies describing the effectiveness of preconception (period before pregnancy and between pregnancies) interventions and risks for adolescent health and reproductive planning on maternal, newborn and child health (MNCH) outcomes. Electronic databases such as PubMed, Cochrane Libraries, Embase, and WHO Regional Databases were searched to identify the studies. We included systematic reviews, experimental and observational studies. Papers were also identified by hand searching references from included studies. No language or date restrictions were applied in the search. The findings were presented at international meeting [11,12] and shared with professionals in the relevant fields of maternal and child health, following which results were updated based on current searches (through end of 2012) and expert opinion. Studies were included if they reported the effectiveness of interventions for promoting adolescent health and reproductive planning on MNCH outcomes. Methodology is described in detail elsewhere [2].

For the studies that met the final inclusion criteria, two review authors abstracted data describing study identifiers and context, study design, intervention specifics and outcome effects into a standardized abstraction sheets. The quality of experimental studies were assessed using Cochrane criteria [13], whereas STROBE guidelines were used to assess the quality of observational studies [14]. We conducted meta-analyses for individual studies and pooled statistics was reported as the odds ratio (OR) and relative risk (RR) between the experimental and control groups with 95% confidence intervals (CI). Mantel–Haenszel pooled RR and corresponding 95% CI were reported or the Der Simonian–Laird pooled RR and corresponding 95% CI where there was an unexplained heterogeneity. All analyses were conducted using the software Review Manager 5.1 [15]. Heterogeneity was quantified by Chi^2^ and I^2^, in situations of high heterogeneity, causes were explored and random effect models were used.

## Results

**Figure 1:**
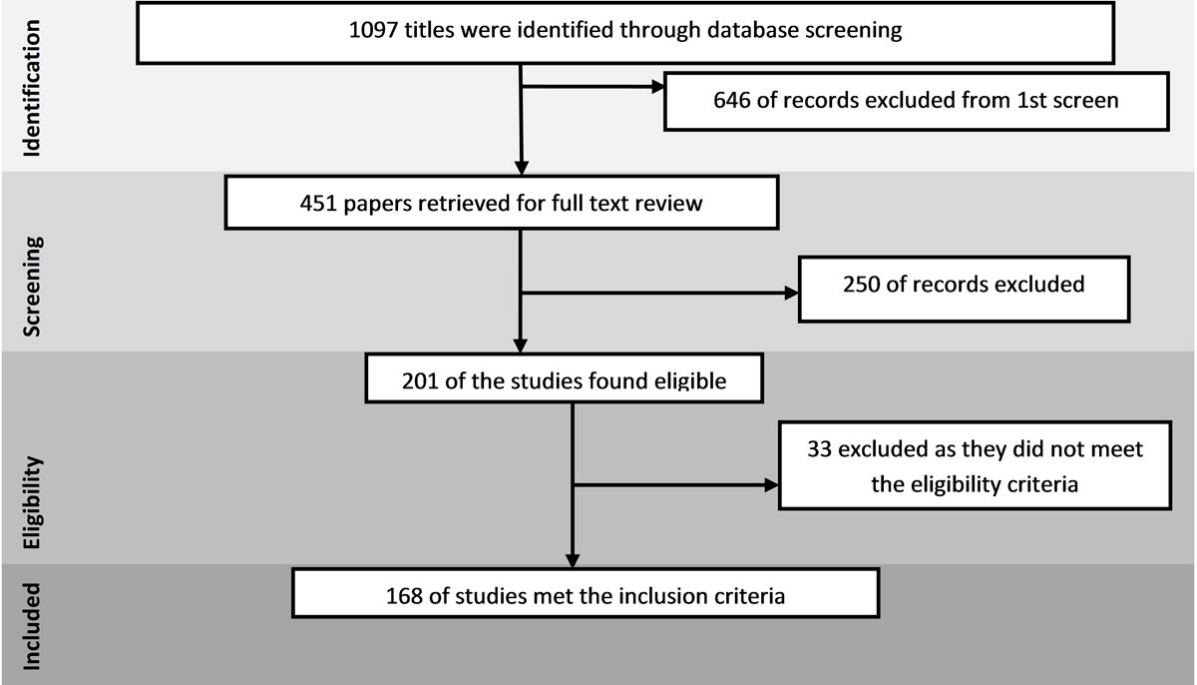
Search flow diagram

The review identified 1097 papers from search in all databases. After the initial title and abstract screening, 451 full texts were reviewed to identify papers which met the inclusion criteria and had the outcomes of our interest. One hundred and sixty eight studies were finally selected for abstraction and analysis (Figure 1). Information related to each included study can be found on the following link: https://globalmotherchildresearch.tghn.org/site_media/media/articles/Preconception_Report.pdf

### Promoting adolescent health

Promoting adolescent health is unquestionably important for a number of reasons [16]. Most importantly, it is the start of reproductive years and it is during the second decade of life that long-term health-related behaviors and coping strategies are formed and fixed; and certain diseases and health problems disproportionately affect adolescents, such as alcohol and substance use, STIs especially HIV/AIDS, and other reproductive health issues. Since adolescents form a significant proportion of the population, especially in LMICs, addressing their health needs is crucial to meet public health and development goals. Improving the health of adolescent girls and young women is central to achieving reductions in global maternal, newborn and childhood mortality and morbidity.

Understanding the risks that adolescents face and examining their behaviors is necessary to develop and scale-up appropriate interventions for this unique population. In many countries, a large percentage of adolescents, both married and unmarried, are sexually experienced and rates of adolescent pregnancy are high [4,17,18]. Determinants of adolescent health and pregnancy vary between countries and regions [19,20] due in part to cultural differences, but there are also common factors (Table 1), notably low levels of contraceptive use, economic and educational disadvantage, lack of information and access to care, and cultural norms that hinder adolescent girls’ independent decision-making [17,18]. In addition, up to 30% of women report that their first sexual experience was coerced- as adolescence is a time where patterns of sexual interaction are being learned, sexual coercion carries profound consequences. In addition to direct reproductive health consequences such as unintended pregnancy or STIs, there are myriad psychological, behavioural and social repercussions, which include subsequent high-risk sex, further experiences of interpersonal violence, and unsafe abortion [21]. In certain cultures, the practice of female genital cutting is pervasive, with 2 million girls under age 15 at risk each year. These girls develop gynaecologic problems, have higher need for obstetric intervention during childbirth, are more likely to experience post-partum haemorrhage and die, and have increased risk of stillbirths and neonatal deaths [22-24].

**Table 1:** Common factors affecting adolescent health & adolescent pregnancy

• Low use of modern contraceptive methods (other than condoms- also use OCPs and withdrawal that are unreliable) • Lower economic and educational status and ambition • Abuse and violence • Alcohol and substance use • Low self-esteem and peer-pressure or lack of decision-making power • Lack of access • Lack of information • Poor relationships and lack of support systems/ family structure (Religion and extracurricular protective) • Multiple sexual partners (especially adolescent males) • Rapid repeat pregnancy • Sexual coercion • Different norms of sexual behavior for girls and boys • Early marriage

Adolescents face complex challenges to their well-being, and adolescent girls are particularly susceptible. Adolescent girls who become pregnant face higher mortality and morbidity, and with the largest cohort of adolescents in history, this is especially worrisome. Adolescent girls dying from pregnancy-related causes accounts for 13% of all maternal deaths. The risk of maternal mortality is twice as high for women aged 15–19 years and five times higher for girls aged 10–14 years compared to women aged 20–29 years. Adolescent girls experience greater frequencies of anemia, complications of labor and delivery, and stillbirths; while their newborns are more likely to be born prematurely, have low birth weight, or die in the first month of life [4,25-27].

Given the underlying circumstances and issues, innovative interventions are needed to mitigate risk, prevent pregnancy and improve health in adolescence. Targeting adolescents and young people with preconception care provides an opportunity to increase knowledge and awareness, and influence the development of healthy behaviors and attitudes early on.

The review identified 9 studies [28-36] and two reviews [37,38]. One school-based prevention programme was shown to significantly reduce psychological, moderate physical and sexual dating violence perpetration, and self-reported decrease in perpetration of violence remained consistent in the long-term [28-30]. A cluster-randomised trial of another school-based intervention showed that the programme had been effective in reducing incidents of physical and emotional abuse and the symptoms of emotional distress for over a year after the programme [31]. One systematic review [37] estimated that on average, multi-component programmes reduced violence by 15% in schools that delivered the programmes compared to those that did not. Another review [38] that examined education programmes for college students found little evidence of the effectiveness of such programmes in preventing such assaults. The literature for intervention programs to prevent coerced sex and dating violence, or reduce their negative effects, largely reports on outcomes associated with a change in the level of knowledge and perceived attitudes post-intervention, but not on behaviours and actual change in the incidence or prevalence of abuse among adolescents. The programs that have been successful incorporate a significant skill-building component, in addition to addressing possible misconceptions of the causes and contributory factors relating to dating aggression.

Studies [32,33] show that the education and empowerment of women is critical for abandoning the practice of female genital mutilation (FGM). Programs carried out over at least a year can increase women’s resolution to not practice FGM on their daughters 1.9-2.6 times post-intervention. The evidence reiterates that interventions to stop the practice of FGM are more successful [34] if they employ a human rights and development approach rather than simply increasing awareness of the consequences [35], use a participatory approach, and involve community leaders including government [36]. Overall the interventions increased the number of community members understanding the consequences of FGM and disapproving of the practice by up to 3 times.

### Preventing first and repeat pregnancy in adolescence

The review identified 33 studies [39-71]. Meta-analyses were conducted of randomized controlled trials that examined any interventions to reduce rates of primary and repeat adolescent pregnancy. Although abstinence-based education has been widely publicized, abstinence-focused sex-education programs insignificantly reduce the risk of pregnancy during adolescence [40,41,72]. Expanded sexual-education programs delivered by adults also did not show an effect in preventing adolescent pregnancy [43,48,51,73], except in one study in Chile [42]. School or health-centre based interventions to promote contraceptive use also had no effect on preventing teenage pregnancy, regardless of whether the intervention involved free provision[74], long-acting or emergency contraception with ease of access [75,76], or peer counselling [49].

**Figure 2:**
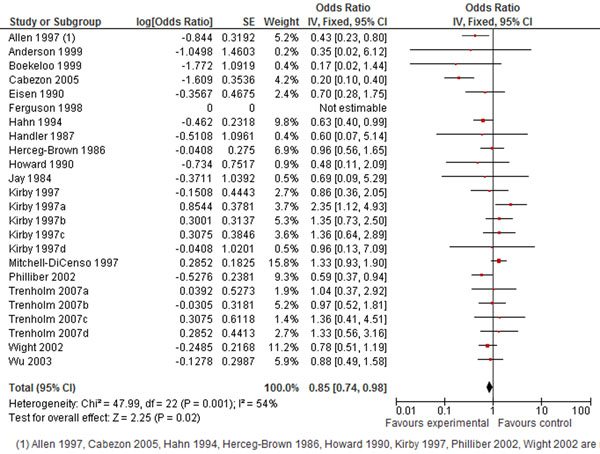
**Prevent teen pregnancy: evidence from controlled trials** Citation to the included studies Allen 1997 [39], Anderson 1999 [40], Boekello 1999 [41], Cabezon 2005 [42], Eison 1990 [43], Ferguson 1998 [44], Hahn 1994 [45], Handle 1987 [46], Herceg-brown 1986 [47], Howard 1990 [48], Jay 1984[49], Kirby 1997 [50], Kirby 1997a [50], Kirby 1997b [50], Kirby 1997c [50], Kirby 1997d [50], Mitchell-dicenso 1997 [51], Philliber 2002 [52], Trenholm 2007a [53], Trenholm 2007b [53], Trenholm 2007c [53], Trenholm 2007d [53], Wight 2002 [54], Wu 2003 [55].

Comprehensive interventions, such as the “Children’s Aid Society Carrera Program” which was carried out in community-centres and provided educational and vocational support, sex education, medical care, sports and arts, free STI testing and condoms are very successful, reducing the risk of teen pregnancy by 41% [52]. Another highly successful program (risk reduction of 57%) focused on youth development through community service, and personal development [39]. Conditional cash transfers for girls to return to school [77] and the use of text messages for education and reminders [78] may be promising as well. The pooled analysis for all interventions to reduce the incidence of adolescent pregnancies showed only a 15% decrease (Figure 2).

Interventions to prevent repeat second pregnancies to teenage mothers include parenting skills training, and encourage teenage mothers to complete their education, regardless of whether they are carried out in health centres, support groups, or during home visits. One particularly successful program (risk reduction 89%) also included comprehensive medical care and referral services for day-care and housing [69]. Another effective program (Second chance club- 84% decreased risk) took a unique approach: it was conducted in high school through individualized case management and group sessions, and focused on school involvement and community outreach, but also provided medical care [62]. Contraceptive hormonal implants successfully prevented repeat teenage pregnancies in one study [79], and contraceptive provision to adolescents might be more successful if implemented in school-based health centres with case management provided by an onsite care provider.

**Figure 3:**
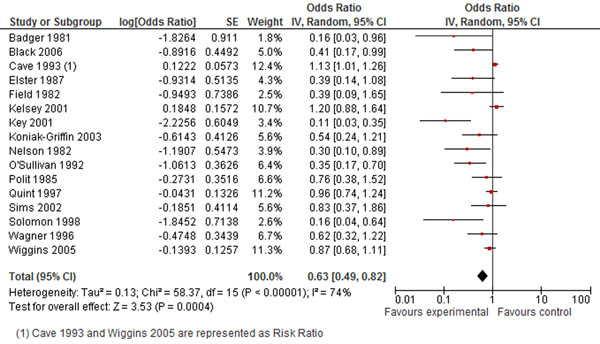
**Prevent REPEAT teen pregnancy: evidence from controlled trials** Citation to the included studies Badger 1981 [56], Black 2006 [57], Cave 1993 [58], Elster 1987 [59], Field 1982 [60], Kelsey 2001 [61], Key 2001 [62], Koniak Griffin 2003 [63], Nelson 1982 [64], O’Sellivan 1992 [65], Polit 1985 [66], Quint 1997 [67], Sims 2002 [68], Solomon 1998 [69], Wagner 1996 [70], Wiggins 2005 [71]

The combined results for all interventions showed a robust effect on decreasing the rate of repeat teenage pregnancies by 37% (Figure 3).

### Birth spacing

Approximately 287,000 women die from causes related to pregnancy and childbirth each year. Of the total number of pregnancies occurring worldwide each year, over 40% are unintended [80]. About one in five pregnancies will end in abortion, nearly half of which are unsafe and cause 47,000 maternal deaths [81]. Many women have abortions because they do not have recourse to family planning services, and thus are unable to plan when or how many children they have. Despite some progress towards achieving Millennium Development Goal 5 to reduce the maternal mortality ratio, a large unmet need for family planning still exists with 222 million women who want the ability to plan their pregnancies not currently using contraception. Preconception care includes reproductive planning, and therefore optimizes birth spacing. This review summarizes the effects of long and short inter-pregnancy intervals on MNCH outcomes, in the attempt to define the ideal interval that can be used to drive counseling and advocacy for appropriate birth spacing as part of Preconception care. The exposure ‘inter-pregnancy’ interval was used to accommodate those intervals where the preceding pregnancy may not have ended in a live birth. Short (<6 months) and long (>60 months) intervals were compared to the ‘ideal’ interval (which studies typically identified as 12-23 months).

Studies have long shown that inter-pregnancy intervals <12 months or >60 months have an adverse effect on maternal and perinatal outcomes [82]. Two recent reviews undertaken by Conde-Agudelo et al. [83,84] to examine the impact of the inter-pregnancy interval on MNCH outcomes found a J-shaped dose-response relationship for perinatal outcomes, but were unable to pool the results for maternal outcomes.

The review identified 21 observational studies [83,85-104]. The meta-analysis found a 32% increase in maternal anaemia for short intervals, but no effect for long intervals. For the same reason, the evidence presented for the effect of short intervals on puerperal endometritis (23% increase), and long intervals on eclampsia (74% increase), third-trimester bleeding (11% increase), and fetal death (18% increase) must be treated with caution.

No significant effects were found for short intervals on third trimester bleeding, postpartum haemorrhage, gestational diabetes mellitus, fetal deaths, preeclampsia or eclampsia. The results for the association of long intervals with postpartum haemorrhage, premature rupture of membranes and gestational diabetes were also not significant.

**Figure 4:**
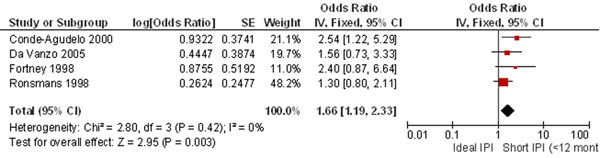
**Short IPI and risk of maternal death: evidence from observational studies** Citation to the included studies Conde-Agudelo 2000 [105], DaVanzo 2005 [107], Fortney 1998 [108], Ronsmans 1998 [109]

We were unable to pool more than two studies for the effect of long intervals on preeclampsia which demonstrated a 74% higher risk; however, the current evidence suggests an increased risk of 60-80% for inter-pregnancy intervals greater than 60 months [84]. Pooling results from four studies showed that short intervals increased the risk of maternal deaths by an alarming 66% (OR 1.66, 95% CI 1.19-2.33) (Figure 4). Short intervals also resulted in a higher frequency (42%) of premature rupture of membranes. Women undergoing a trial of labour after a short interval were thrice as likely to suffer uterine rupture (OR 3.04, 95% CI 1.91-4.85).

The meta-analysis found a significantly increased risk of adverse perinatal outcomes with inappropriate pregnancy spacing. Short and long intervals increased the risk of preterm birth (OR 1.45 and 1.21 respectively), low birth weight (OR 1.65 and 1.37 respectively), and small-for-gestational age (OR 1.17 and 1.18 respectively). Conde-Agudelo & Belizan did not pool the estimates for stillbirths and neonatal death; however the meta-analysis shows an increase in stillbirths for short intervals (OR 1.42, 95% CI 1.09-1.86) and no significant effect for long intervals [105]. Conversely, there was an increased risk of neonatal deaths with long (OR 1.15, 95% CI 1.06-1.25), but not short intervals. A recent study [106] found that both short and long inter-pregnancy intervals elevate the odds of congenital defects (OR 1.15, 95% CI 1.03-1.28 and OR 1.15, 95% CI 1.04-1.26, respectively).

A WHO technical consultation [110] was held in 2005 to decide, based on the research evidence, what constitutes the ideal inter-pregnancy interval. Noting the effects of short intervals (<12 months) and long intervals (>60 months) on maternal and perinatal outcomes, especially mortality, experts recommended a space of 18-24 months after a live birth. A recommendation for pregnancy spacing of 24 months would coincide with the optimal duration of breastfeeding, conferring added nutritional benefit in early childhood.

### Reproductive planning after abortion

Many women resort to induced abortion as a desperate means of reproductive planning after an unintended pregnancy has occurred. Lack of access to services and the illegality or social unacceptability of abortion in many countries means that women often resort to crude and dangerous means to end a pregnancy [111]. Complications of unsafe abortion include incomplete abortion, hemorrhage, sepsis, uterine perforation, intra-abdominal injury, psychological trauma, infertility, reproductive tract infections, and maternal death. 21.6 million women are estimated to undergo unsafe abortions, that could be avoided through access to family planning services, and safe abortion care.Safe abortion care has the potential to save 70,000 women and prevent 5 million disabilities annually [7]. Care after an abortion includes emergency treatment of abortion complications, and provision of (or referral to) other reproductive health and counseling services [112].

The review identified 20 studies [112-131]. The included studies focused on whether women received post abortion contraceptive counseling and a contraceptive method before leaving the health facility, as well as the use of contraception at follow-up. Most studies were pre-post design and unfortunately very few reported subsequent outcomes of interest such as the incidence of repeat abortions, pregnancy complications, or length of interval before the next pregnancy. The methods of improving post-abortion care included training healthcare providers, providing equipment and contraceptive methods, counseling partners, service reorganization and collaboration, improved follow up, linkage with other reproductive health services, and rarely increasing community awareness.

The greatest improvement in women’s uptake of contraception after an abortion was seen with 2 interventions: provision of emergency treatment, contraceptive counseling, and community-service provider partnerships from 2% to 86.6% in three years; and training of providers, counseling, free contraception and follow-up resulted in 96% uptake at intervention site versus 5% at control site. The second intervention also showed that women receiving care were 3.38 times less likely to have an unplanned pregnancy, and 8% fewer repeat abortions. The least improvement (14%) was seen in one study [116] with free contraceptive provision. This should be interpreted with caution because repeat abortions among women receiving the intervention decreased to half the rate in the general population. Counseling also creates opportunities to involve women’s partners [118] by increasing the likelihood (OR 1.6) that they will support contraceptive uptake; women who receive partners’ support are almost 6 times more likely to use contraception.

### Advanced maternal age

Couples’ decisions regarding childbearing are strongly influenced by sociocultural and economic factors. There is a growing trend towards delayed childbearing as more young people pursue higher education and desire financial independence before they start a family. Higher divorce rates and the lack of a strong support system also play a role in the decision to become a parent during later reproductive years [132]. There is a general understanding that as fertility declines, couples may have more difficulty conceiving at a later age, and that the risk of triploid disorders is increased. In addition, there may be long-term consequences for parenting behaviors [133-135].

The review compared the MNCH outcomes for women of any age over 35 years, with women any age between 20 and 35 years. Most of the evidence came from risk-aversion studies. Since the association of advanced maternal age and chromosomal aberrations is already well established, this data was not used.

The review identified 60 observational studies [136-195]. Pregnancy at an early age and during the later reproductive years may both influence maternal and child health outcomes. A significantly increased risk of Caesarean delivery was found with advanced maternal age (RR 1.72, 95% CI 1.59-1.85). However, since this estimate includes elective C-sections, the risk might simply be attributable to obstetricians, and women themselves, exercising more caution in gravidas over age 35. Although hypertension was defined differently in various studies, the risk of hypertension during pregnancy increased 3 times for women of advanced maternal age. We also found an elevated, but insignificant, risk of pre-eclampsia (0R 2.06, reduced to 1.60 when a study with very wide confidence intervals was removed from the analysis), which was taken to be a more sensitive indicator than hypertension overall in this population. The risk for antepartum hemorrhage, specifically that due to placenta previa, was significant, being approximately 3 times higher in women of advanced maternal age. The risk for maternal gestational diabetes was also significantly 3 times higher, whereas the risk for pre-gestational diabetes was increased six-fold

**Figure 5:**
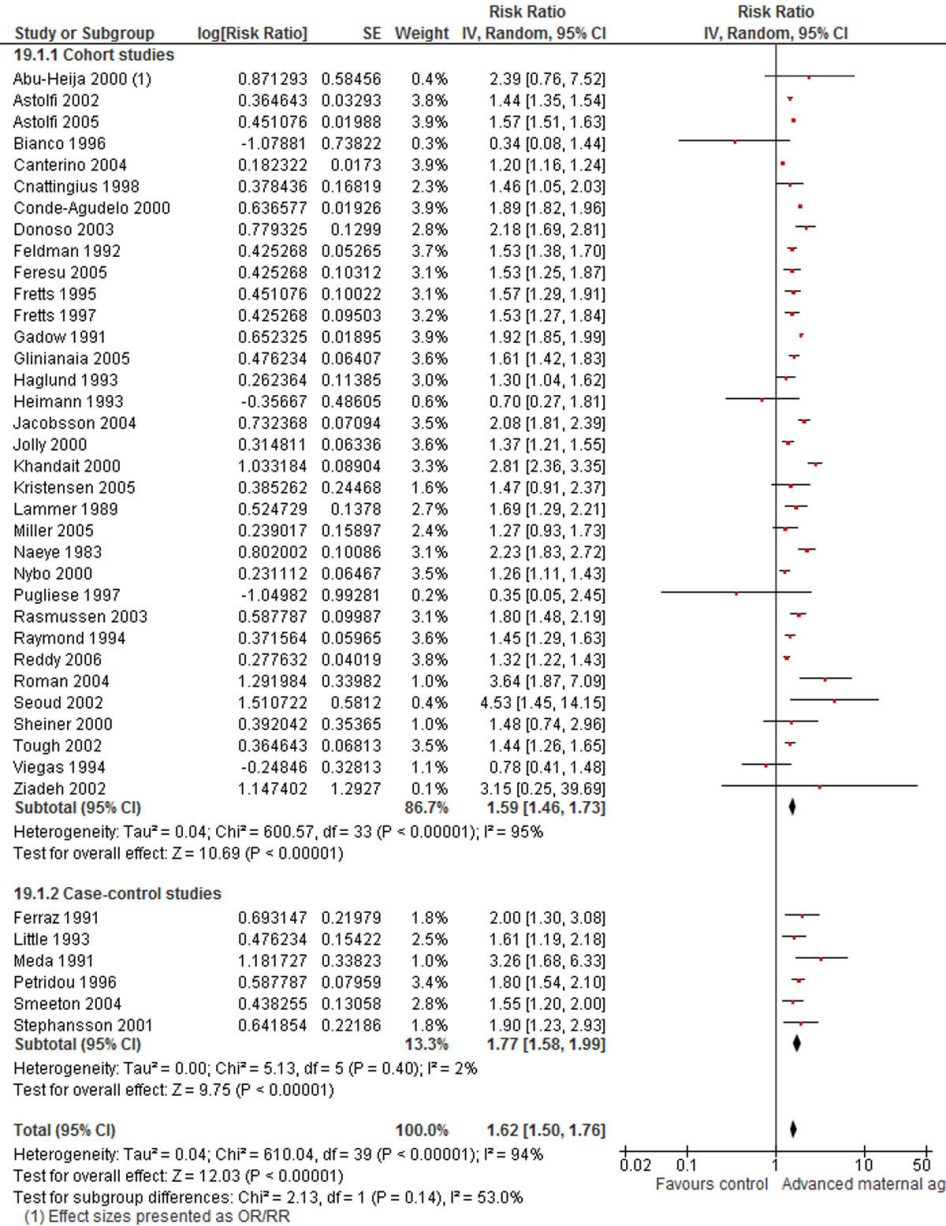
**Advanced maternal age and risk of stillbirths: evidence from observational studies** Citation to the included studies: Abu-Heija 2000 [161], Astolfi 2002 [162], Astolfi 2005 [163], Blanco 1996 [138], Canterino 2004 [164], Cnattingius 1998 [143], Conde-Agudelo 2000 [196], Donoso 2003[166], Feldman 1992 [167], Feresu 2005 [168], Fretts 1995 [169], Fretts 1997 [170], Gadow 1991 [171], Gliniania 2005 [172], Haglund 1993 [173], Heimann 1993 [174], Jacobsson 2004 [147], Jolly 2000 [148], Khandait 2002 [175], Kristensen 2005 [176], Lammer 1989 [177], Miller 2005 [153], Naeye 1983 [178], Nybo 2000 [179], Pugliese 1997 [180], Rasmussen 2003 [181], Raymond 1994 [182], Reddy 2006 [183], Roman 2004 [184], Seoud 2002 [185], Sheiner 2000 [186], Tough 2002 [187], Viegas 1884 [188], Ziadeh 2002 [189], Ferraz 1991 [190], Little 1993 [191], Meda 1991 [192], Petridou 1996 [193], Smeeton 2004 [194], Stephansson 2001 [197]

We also found a higher (62%) risk of stillbirths with delayed childbearing, which is statistically significant and was derived from a total of 40 cohort and case-control studies (Figure 5). Although fewer studies were included in the meta-analyses for advanced maternal age and the risk of perinatal death (increased risk by 44%; 95% CI: 1.10-1.89), preterm birth (increased risk by 29% 95% CI: 1.14-1.46) and low birth weight (increased risk by 61%; 95% CI: 1.16-2.24), the analysis yielded significant effects for maternal age greater than 35 years on each of these outcomes.

### Genetic counselling

Genetic counselling involves diagnosis, information provision/explanations, and discussion of possible options. There may be debate about what method may work best and what time may be appropriate for such an intervention to have the maximum effects. Rowley et al. reported that a patient-structured counselling method, designed to minimize negative psychological effects via discussion of feelings, was equivalent to conventional and programmed methods in terms of learning or attitude change.

Despite an extensive search we did not find studies the reported the impact of genetic counseling on maternal, newborn and child health. We only came across literature related to the attitudes and perception of couples regarding the provision of these services and the general attitude of physicians towards genetic counseling and screening in the preconception period. Our search yielded data only pertaining to cystic fibrosis, fragile X, Tay Sachs and thalassemia.

The review identified 23 studies [198-220]. Studies on cystic fibrosis generally addressed the attitudes and perceptions of couples regarding the possibility of preconception screening. In a study where couples were asked if they would participate in a preconception screening for CF, majority replied in the affirmative [221]. However another study [204] reported a 74% acceptance rate of free preconception screening for common genetic disorders which only translated into a 2% submission rate of their blood samples. It was reported that a majority perceived no impact of carrier testing on their relationship status with their partner; this could generally be taken as a positive sign for support. Another study reported that carriers who had undergone had a poorer perception of their health 3 years post-testing, as compared to non-carriers [198,199]. Attitudes of health professionals regarding preconception CF carrier screening varied considerably. Greatest support to the notion was given by General Practitioners [200-202]; however this attitude was not translated into practice [203]. among the mode of delivery of information for population based screening, studies showed that the uptake rates were higher if the written information was given [204], if screening was offered in person by a health professional [205,206] and if immediate testing was offered [206,207].

A recent review for screening for Fragile X, found no trials to show whether offering the test to everyone is worthwhile. However studies have identified a positive attitude towards preconception screening amongst women planning a pregnancy as well as those in the general community [222] and amongst physicians [20].

Studies for premarital screening for those with hemoglobinopathies showed varying results with most reporting couples still proceeding with their marriage plans despite positive test results and counseling [208-211]; some still showed a positive effect of such programs with couples paying heed to the advice [212,213]. In the event of an ‘inter-carriers’ union, many of those who had received genetic counseling, whether in school [214-216] or elsewhere [217], sought prenatal diagnosis.

With regards to an actual effect on the disease prevalence post screening interventions, data is only available from national screening programs for thalassemia. While the genetic screening program for Thailand was unsuccessful, that of Iran deserves to be applauded. This integrated premarital screening program led to a 70% reduction in thalassemia birth rate. At risk couples were referred for counseling and were subsequently followed. Such national thalassemia prevention programs and obligatory premarital screening programs have drastically reduced thalassemia rates in these areas [212,218-220]

We found limited evidence identifying the effectiveness of any genetic screening and counseling, provided in the preconception period, in dealing with outcomes related to pregnancies. We found that couples are generally receptive to such services. This fact, and the example provided by Iran’s screening program for thalassemia, needs to be utilized by health policy makers in devising comprehensive genetic counseling to all couples planning a pregnancy and genetic screening services keeping in mind the regional prevalence of genetic disorders.

## Discussion

In spite of increased contraceptive coverage, many women continue to become pregnant when they do not intend to. Women may not have easy access to effective modern methods of contraception or may not use it correctly; rates of teenage pregnancy continue to be high across the world; simultaneously many women are choosing to delay initiation of childbearing; women also continue to suffer the consequences of coerced sex and intimate partner violence- these are just a few of the complex factors that lead to unintended pregnancy, and deleterious inter-pregnancy intervals. In addition to undergoing unsafe abortions, women who have unplanned pregnancies are also less likely to seek prenatal care, are more likely to engage in risky behaviour such as alcohol use and smoking, and are more likely to become depressed. When women carry these pregnancies to term, they are less likely to breastfeed or continue breastfeeding, and their children are more likely to be neglected and undernourished [223]. It seems logical that a substantial proportion of these adverse outcomes of unplanned pregnancies could be averted by bolstering efforts to meet the current contraceptive need. Comprehensive interventions that address communities, sexual and reproductive health services, contraceptive provision and school-based education; and youth development programs which promote personal development, completion of education, and community service are highly effective in preventing teenage pregnancies. The success of combining multiple interventions, especially contraception with education, has also been reported in a recent Cochrane review [224]. Systematic reviews by DiCenso et al. and Corcoran & Pillai 2007 [225,226] confirm that the cumulative evidence for effective teenage pregnancy prevention programs is modest since programs differ in context and content. For adolescents who are already mothers, parental skills training and encouraging them to complete their education, while providing them with medical care, prevents repeat pregnancy during adolescence.

The evidence demonstrates that short inter-pregnancy intervals increases the risk of adverse perinatal outcomes, including preterm birth, low birth weight, stillbirths, maternal mortality and early neonatal deaths; while long inter-pregnancy interval heightens the risk of preeclampsia, which is a cause of maternal mortality. Some inconsistencies between the results of this review and previous work, such as the association of short inter-pregnancy intervals with anaemia and puerperal endometritis, or long inter-pregnancy intervals with eclampsia, third-trimester bleeding and fetal death, may be explained because we pooled only two studies, one of which included only women whose preceding pregnancy ended in an abortion [89]. Although the interaction between contraceptive use and pregnancy intention is complex [227,228], recent reviews have found a trend toward increasing pregnancy intendedness and appropriate intervals with the use of contraception [229,230], however this effect does not extend to advance provision of emergency contraception [231]. Better understanding and measures of pregnancy intention, timing and wantedness are needed to increase contraceptive uptake and use [232]. Research has shown that investment in meeting the need for modern contraception in developing countries, where the burden of maternal and child mortality and morbidity is highest, would be highly cost-effective. Further, these two interventions would greatly contribute to improving adolescent health, women’s health and health equity overall. Birth spacing is itself an intervention with evidence to support its effect on maternal mortality and important perinatal outcomes, yet while it is strongly recommended, there remains a need for development of strategies to promote birth spacing before first pregnancy, during pregnancy and between pregnancies

The studies overall demonstrate that post abortion care successfully increases contraceptive uptake among approximately 90% of women who receive it, as well as their partners’ support and participation in family planning, however more evidence is needed to show whether this translates to fewer unintended pregnancies and fewer abortions.

Previous reviews have suggested that with advanced maternal age, the presence of comorbidities -especially diabetes and hypertension- increases, and this might largely be responsible for the pregnancy outcomes in women who delay childbearing. Schoen & Rosen 2009 [233] also reported significantly increased risks of maternal complications in women who delayed pregnancy till their later reproductive years. Although the evidence shows an inherently greater risk with pregnancy at advanced maternal age, the social stimulus behind this trend might prove difficult to change, especially with the advent of assisted reproductive technology. Counselling is especially important for women with pre-existing medical conditions, such as diabetes and hypertension, since these contribute to excess morbidity during gestation. Research might provide further insight into the mechanisms of risk including possible confounders such as parity and method of conception, and possible interventions such as pre-implantation genetic diagnosis might become more accessible. At present, public health interventions can increase awareness regarding advanced parental age, allowing couples to weigh the risks and benefits of delaying childbearing [234], and quality antenatal care should be provided to those who become pregnant later.

While there was a significant impact of delaying childbearing on selected MNCH outcomes, these estimates must be interpreted with caution [235], since included studies considered different age cut-offs as “advanced” and comparison groups, and many studies did not explicitly separate conceptions through assisted reproduction. Perhaps as this population grows in number, larger prospective studies that control for confounders will substantiate whether advanced maternal age really is an independent risk factor for poor MNCH outcomes. Although the evidence shows an inherently greater risk with pregnancy at advanced maternal age, the social stimulus behind this trend might prove difficult to change, especially with the advent of assisted reproductive technology. Counselling is especially important for women with pre-existing medical conditions, such as diabetes and hypertension, since these contribute to excess morbidity during gestation. Research might provide further insight into the mechanisms of risk including possible confounders such as parity and method of conception, and possible interventions such as pre-implantation genetic diagnosis might become more accessible. At present, public health interventions can increase awareness regarding advanced parental age, allowing couples to weigh the risks and benefits of delaying childbearing [234], and quality antenatal care should be provided to those who become pregnant later.

Although limited evidence [218] was found on genetic screening and counseling, it was reported that couples are generally receptive to such services. Therefore, comprehensive genetic counseling to all couples planning a pregnancy and genetic screening services to women is worthy particularly for those where the regional prevalence of genetic disorders are high. Providing preconception care that incorporates reproductive planning and genetic counseling can positively influence health in adolescents, young women and couples, and avert many negative MNCH outcomes over the next generation.

## Conclusion

Early marriage, risky sexual behaviors, delayed childbearing, and lack of access to contraception or safe abortion care mean that girls/women experience disproportionately high rates of intrapartum complications, stillbirths, neonatal deaths, prematurity and low birth weight. Among the risks and interventions reviewed, two are strongly recommended: programs to prevent first and repeat pregnancy during adolescence, and strategies to promote appropriate spacing between pregnancies through increasing uptake and consistent use of effective contraception. The successful implementation can be achieved through programs such as personal development and community service; school-based sexual and reproductive health education; and contraceptive provision. Whereas some promising interventions such as contraceptive counseling integrated into safe abortion care and conditional cash transfers to keep adolescent girls in school need further evaluation, increasing delivery and coverage of proven interventions through preconception care should now become a priority. Programs to prevent adolescent pregnancy can be adapted to different contexts, and scaled up in those contexts where they have previously been piloted. Promoting reproductive planning on a wider scale is closely interlinked with the reliable provision of effective contraception, however innovative strategies will need to be devised, or existing strategies to promote maternal, newborn and child health (such as community-based health workers and peer educators) may be expanded, to encourage girls and women to consider how they wish to plan their family.

## Competing interests

We do not have any financial or non-financial competing interests for this review.

## Peer review

Peer review reports are included in additional file 1.

## Supplementary Material

Additional file 1 Peer review reports. Click here for file

## Declarations

This article has been published as part of *Reproductive Health* Volume 11 Supplement 2, 2014: Preconception interventions. The full contents of the supplement are available online at http://www.reproductive-health-journal.com/supplements/11/S3. Publication charges for this collection were funded by the Partnership for Maternal, Newborn & Child Health (PMNCH).
