# Supplementary material for: Preconception care: promoting reproductive planning
Source: Reprod Health. 2014 Sep 26;11(Suppl 3):S2. doi: 10.1186/1742-4755-11-S3-S2 (PMC4196558; doi:10.1186/1742-4755-11-S3-S2)
Supplement: Additional file 1 — Peer review reports. [file 1742-4755-11-S3-S2-S1.pdf]

Referee's comments to the authors– this sheet WILL be seen by the author(s) and published with the article

Title	**Preconception care: promoting adolescent health and reproductive planning**
Author(s)	Sohni V Dean, Zohra S Lassi, Ayesha M Imam, Zulfiqar A Bhutta
Referee's name	Anne Austin

When assessing the work, please consider the following points, where applicable:

1. Is the question posed by the authors new and well defined?
2. Are the methods appropriate and well described, and are sufficient details provided to replicate the work?
3. Are the data sound and well controlled?
4. Does the manuscript adhere to the relevant standards for reporting and data deposition?
5. Are the discussion and conclusions well balanced and adequately supported by the data?
6. Do the title and abstract accurately convey what has been found?
7. Is the writing acceptable?

Please make your report as constructive and detailed as possible in your comments so that authors have the opportunity to overcome any serious deficiencies that you find and please also divide your comments into the following categories:

- Major Compulsory Revisions (which the author must respond to before a decision on publication can be reached)
- Minor Essential Revisions (such as missing labels on figures, or the wrong use of a term, which the author can be trusted to correct)
- Discretionary Revisions (which are recommendations for improvement but which the author can choose to ignore)

Where possible please supply references to substantiate your comments.

When referring to the manuscript please provide specific page and paragraph citations where appropriate.

General comments:
I feel that this paper incorporates a few too many themes, and perhaps could be split into multiple papers. For example:

1) Interventions to promote adolescent health, through preconception care
2) The impact of birth spacing on maternal health
3) Pregnancy risks of advanced maternal age and the association with genetic counseling

As presented now, it is a bit disjointed. The combination of risk factors and interventions could be more clearly delineated and the conclusion focuses mainly on interventions.

Major compulsory revisions:

Methods
The methods section needs to be expanded to clarify the search and inclusion criteria- i.e beyond "standard methods". There also needs to be greater explanation (i.e fixed effects model) on the methods for meta-analysis**.**

(continue on the next sheet)

Continued:

Preventing first and repeat pregnancy in Adolescence: (intervention)
Some of the articles included in the meta-analysis for the prevention of teen pregnancy did not include pregnancy as an outcome. For example, Boekello looked at STI/HIV infection and risky sexual behavior, and Hahn was looking at interventions to prevent violence.

Birth Spacing: (intervention)
I am not sure how we went from preconception care to birth spacing. The link to preconception care, which includes family planning, and therefore optimizes birth spacing, should be more explicit. This meta-analysis confirms many of the findings from earlier meta-analyses, but I am not sure of the additional value this adds to the evidence base.

The last paragraph of the birth spacing section is a bit of an awkward amalgamation of unintended pregnancy, unmet need, unplanned pregnancy and contraceptive use- the pathways between each of these issues needs to be clearly articulated.

Birth spacing, is an intervention that can be implemented during the preconception phase as well as part of an ANC package, or PNC counseling.

Advanced maternal age: (risk)
The MNCH outcomes found in this analysis are consistent with available evidence. Are the authors arguing that preconception interventions need to be tailored to women of advanced maternal age, due to the increased risk of advanced maternal age on MNCH outcomes? If so, this should be explicit. Unlike earlier sections of the paper, this section looks at the link between advanced maternal age and MNCH outcomes, not at the evidence of interventions targeted at this group- it doesn't quite fit in the paper.

Genetic Counselling: (intervention)
In paragraph two, you should state what the outcome of interest is.
In many cases, genetic counselling often leads couples to select to abort an unviable child. It is not surprising that post-screening programs lead to a 70% reduction in Thalassemia birth rates.
Is there any empirical evidence of this?

Minor essential revisions:

Figure 4: Short IPI and risk of maternal death is missing

Discretionary revisions:
The section Reproductive planning after a pregnancy loss should probably be re-titled post-abortion interventions to improve maternal health- as it specifically focused on induced abortion, rather than miscarriage.

Referee's comments to the authors– this sheet WILL be seen by the author(s) and published with the article

Title	**Preconception care: promoting adolescent health and reproductive planning**
Author(s)	Sohni V Dean, Zohra S Lassi, Ayesha M Imam, Zulfiqar A Bhutta
Referee's name	Khadija Nuzhat Humayun

When assessing the work, please consider the following points, where applicable:

1. Is the question posed by the authors new and well defined?
2. Are the methods appropriate and well described, and are sufficient details provided to replicate the work?
3. Are the data sound and well controlled?
4. Does the manuscript adhere to the relevant standards for reporting and data deposition?
5. Are the discussion and conclusions well balanced and adequately supported by the data?
6. Do the title and abstract accurately convey what has been found?
7. Is the writing acceptable?

Please make your report as constructive and detailed as possible in your comments so that authors have the opportunity to overcome any serious deficiencies that you find and please also divide your comments into the following categories:

- Major Compulsory Revisions (which the author must respond to before a decision on publication can be reached)
- Minor Essential Revisions (such as missing labels on figures, or the wrong use of a term, which the author can be trusted to correct)
- Discretionary Revisions (which are recommendations for improvement but which the author can choose to ignore)

Where possible please supply references to substantiate your comments.

When referring to the manuscript please provide specific page and paragraph citations where appropriate.

General comments:
Abstract:
No comments

Major compulsory revisions:

Minor essential revisions:

- Please add the word years after 20-25
- Last paragraph of page 5 the abbreviation FGM is used multiple times and full word is missing please add that.
- Page 10, second paragraph "The review identified [118-137]." This sentence seems incomplete.
- Page 10, third paragraph 3rd line should be 2% to 86.6%.
- Page 11, first paragraph the message is not very clear. Please elobrate further.
- Figure 4 is incomprehensible in the PDF file please modify it

(continue on the next sheet)

Continued:

Discretionary revisions:

- Methods section can be more detailed to elucidate how the search was conducted.
- Authors can relook at genetic counseling section although it is important but is not directly related to the recommendations and conclusion they are making. They may consider revising this part.

Referee's comments to the authors– this sheet WILL be seen by the author(s) and published with the article

Title	**Preconception care: promoting adolescent health and reproductive planning**
Author(s)	Sohni V Dean, Zohra S Lassi, Ayesha M Imam, Zulfiqar A Bhutta
Referee's name	Rehana Salam

When assessing the work, please consider the following points, where applicable:

1. Is the question posed by the authors new and well defined?
2. Are the methods appropriate and well described, and are sufficient details provided to replicate the work?
3. Are the data sound and well controlled?
4. Does the manuscript adhere to the relevant standards for reporting and data deposition?
5. Are the discussion and conclusions well balanced and adequately supported by the data?
6. Do the title and abstract accurately convey what has been found?
7. Is the writing acceptable?

Please make your report as constructive and detailed as possible in your comments so that authors have the opportunity to overcome any serious deficiencies that you find and please also divide your comments into the following categories:

- Major Compulsory Revisions (which the author must respond to before a decision on publication can be reached)
- Minor Essential Revisions (such as missing labels on figures, or the wrong use of a term, which the author can be trusted to correct)
- Discretionary Revisions (which are recommendations for improvement but which the author can choose to ignore)

Where possible please supply references to substantiate your comments.
When referring to the manuscript please provide specific page and paragraph citations where appropriate.

General comments:
A huge body of knowledge but not presented clearly.

Major compulsory revisions:
The title suggests that the authors will evaluate **adolescent health and reproductive planning**, while the paper doesn't sufficiently cover **"adolescent health"** as adolescent health in itself is a vast domain. Suggest changing the title to reflect what was done in the review.

Introduction seems to be hastily written and lacks flow

In introduction, 1st paragraph, "**For 1.2 billion adolescents…."** Needs to be updated with the recent data

Please define briefly "preconception care" in the background to give readers a clearer idea of what the review is about

In methods section **"Standard criteria were applied to determine the quality of the evidence, and standard methods for meta-analyses of quantitative studies were used."** Please mention which criteria..

In methods section **"The findings were presented at international meetings and shared with professionals in the relevant fields of maternal and child health, following which results were updated based on current searches (through end of 2012) and expert opinion."** What changes were made after the expert consultation? Were the changes significant enough to be mentioned in the paper??

The methods section is not clear. It presents the paper as a systematic review and meta-analysis, however the results sections mentions findings from existing reviews… please describe the methodology more clearly

The methods section should also clarify that the authors reviewed both the **interventions** and the **risks**

Results section should not include parts of introduction/ background. Parts of results are already mentioned in the background. Please remove

In the results section, Suggest discussing the concordance and discordance with other reviews in the discussion section

The results section of the abstract needs to be re-written as it clearly misses out on many interventions reviewed.

Minor essential revisions:

The paper starts with the heading **'Why adolescent health and reproductive planning are important from the preconception perspective'.** This looks more like a statement then a heading. Can be replaced with "**Background" OR "introduction"** since the title already gives an idea that it relates to preconception care.

In introduction 1st paragraph, "**Adolescent girls and young women are particularly vulnerable, facing interpersonal violence, coerced intercourse, sexually-transmitted infections especially HIV, undernutrition or obesity and their health consequences; besides the social pressures that prevent them from completing their education and force them into early marriages and childbearing."** very long sentence, suggest clarifying and shortening

In Introduction, last paragraph, "**Millions of women and children, and future generations will benefit if we invest in Preconception care to improve adolescent health and encourage reproductive planning."** Looks like a concluding statement. Suggest removing it

In Introduction, last paragraph**, "This paper presents the findings of a systematic review that was undertaken to consolidate the evidence for risks and interventions relating to this area. It begins**….." needs to be re-written, not clear

Can't see figure 4

In the results section, "reproductive planning after a pregnancy lost", 2nd paragraph **"The review identified ..". please complete the sentence**

Referee's comments to the authors– this sheet WILL be seen by the author(s) and published with the article

Title	**Preconception care: promoting adolescent health and reproductive planning**
Author(s)	Sohni V Dean, Zohra S Lassi, Ayesha M Imam, Zulfiqar A Bhutta
Referee's name	Anne Austin

When assessing the work, please consider the following points, where applicable:

1. Is the question posed by the authors new and well defined?
2. Are the methods appropriate and well described, and are sufficient details provided to replicate the work?
3. Are the data sound and well controlled?
4. Does the manuscript adhere to the relevant standards for reporting and data deposition?
5. Are the discussion and conclusions well balanced and adequately supported by the data?
6. Do the title and abstract accurately convey what has been found?
7. Is the writing acceptable?

Please make your report as constructive and detailed as possible in your comments so that authors have the opportunity to overcome any serious deficiencies that you find and please also divide your comments into the following categories:

- Major Compulsory Revisions (which the author must respond to before a decision on publication can be reached)
- Minor Essential Revisions (such as missing labels on figures, or the wrong use of a term, which the author can be trusted to correct)
- Discretionary Revisions (which are recommendations for improvement but which the author can choose to ignore)

Where possible please supply references to substantiate your comments.

When referring to the manuscript please provide specific page and paragraph citations where appropriate.

General comments:
I feel that this paper incorporates a few too many themes, and perhaps could be split into multiple papers. For example:
1) Interventions to promote adolescent health, through preconception care
2) The impact of birth spacing on maternal health
3) Pregnancy risks of advanced maternal age and the association with genetic counseling
As presented now, it is a bit disjointed. The combination of risk factors and interventions could be more clearly delineated and the conclusion focuses mainly on interventions.
There were number of Preconception interventions and risks that we grouped under different themes (such as adolescent health, nutritional health, infectious diseases, chronic diseases etc). In this paper we included all interventions that are relevant to adolescent health and reproductive planning.

Regarding the second point. We have now explicitly mentioned whether the evidence is coming from observational studies or experimental studies in the text and in the plots. Conclusions are mainly based on evidence from experimental studies, while few from observational studies (for risks where trials were not suitable).

The background section addresses why adolescent health and reproductive planning are fundamental to preconception care. These topics are presented together because reproductive planning should be a discussion that begins in adolescence.

Major compulsory revisions:
Methods
The methods section needs to be expanded to clarify the search and inclusion criteria- i.e beyond "standard methods". There also needs to be greater explanation (i.e fixed effects model) on the methods for meta-analysis**.**
We have now added a detailed section on methods on pages 3-4.

Preventing first and repeat pregnancy in Adolescence: (intervention)
Some of the articles included in the meta-analysis for the prevention of teen pregnancy did not include pregnancy as an outcome. For example, Boekello looked at STI/HIV infection and risky sexual behavior, and Hahn was looking at interventions to prevent violence.
Although pregnancy prevention was not the primary outcome reported in these studies, pregnancy was reported as an outcome in both the intervention and control groups, therefore these were included in the meta-analysis.

Birth Spacing: (intervention)
I am not sure how we went from preconception care to birth spacing. The link to preconception care, which includes family planning, and therefore optimizes birth spacing, should be more explicit. This meta-analysis confirms many of the findings from earlier meta-analyses, but I am not sure of the additional value this adds to the evidence base.
The definition of preconception care covers preconception and interconception care. We have, therefore, included interventions for purely preconception and those which can be advised and encouraged during interconception.

The last paragraph of the birth spacing section is a bit of an awkward amalgamation of unintended pregnancy, unmet need, unplanned pregnancy and contraceptive use- the pathways between each of these issues needs to be clearly articulated.Birth spacing, is an intervention that can be implemented during the preconception phase as well as part of an ANC package, or PNC counseling.
Revisited the section. Also added the phases when these interventions should be reinforced. "Birth spacing is itself an intervention with evidence to support its effect on maternal mortality and important perinatal outcomes, yet while it is strongly recommended, there remains a need for development of strategies to promote birth spacing before first pregnancy, during pregnancy and between pregnancies"

Advanced maternal age: (risk)
The MNCH outcomes found in this analysis are consistent with available evidence. Are the authors arguing that preconception interventions need to be tailored to women of advanced maternal age, due to the increased risk of advanced maternal age on MNCH outcomes? If so, this should be explicit. Unlike earlier sections of the paper, this section looks at the link between advanced maternal age and MNCH outcomes, not at the evidence of interventions targeted at this group- it doesn't quite fit in the paper.
As mentioned in the last paragraph of the introduction and methods section that this review is looking at the evidence for risks and interventions relating to preconception area. Maternal age at first pregnancy is an important area which comes under the reproductive planning. It was therefore, important to highlight the risk associated with advance maternal age.

Genetic Counselling: (intervention)
In paragraph two, you should state what the outcome of interest is. Added
In many cases, genetic counselling often leads couples to select to abort an unviable child. It is not surprising that post-screening programs lead to a 70% reduction in Thalassemia birth rates. Is there any empirical evidence of this? No, and it is therefore mentioned in as "With regards to an actual effect on the disease prevalence post screening interventions, data is only available from national screening programs for thalassemia"

Minor essential revisions:
Figure 4: Short IPI and risk of maternal death is missing. It is on Page 9

Discretionary revisions:
The section Reproductive planning after a pregnancy loss should probably be re-titled post-abortion interventions to improve maternal health- as it specifically focused on induced abortion, rather than miscarriage. Renamed as Reproductive planning after abortion

Referee's comments to the authors– this sheet WILL be seen by the author(s) and published with the article

Title	**Preconception care: promoting adolescent health and reproductive planning**
Author(s)	Sohni V Dean, Zohra S Lassi, Ayesha M Imam, Zulfiqar A Bhutta
Referee's name	Khadija Nuzhat Humayun

When assessing the work, please consider the following points, where applicable:

1. Is the question posed by the authors new and well defined?
2. Are the methods appropriate and well described, and are sufficient details provided to replicate the work?
3. Are the data sound and well controlled?
4. Does the manuscript adhere to the relevant standards for reporting and data deposition?
5. Are the discussion and conclusions well balanced and adequately supported by the data?
6. Do the title and abstract accurately convey what has been found?
7. Is the writing acceptable?

Please make your report as constructive and detailed as possible in your comments so that authors have the opportunity to overcome any serious deficiencies that you find and please also divide your comments into the following categories:

- Major Compulsory Revisions (which the author must respond to before a decision on publication can be reached)
- Minor Essential Revisions (such as missing labels on figures, or the wrong use of a term, which the author can be trusted to correct)
- Discretionary Revisions (which are recommendations for improvement but which the author can choose to ignore)

Where possible please supply references to substantiate your comments.

When referring to the manuscript please provide specific page and paragraph citations where appropriate.

General comments:
Abstract:
No comments

Major compulsory revisions:

Minor essential revisions:

- Please add the word years after 20-25 added.
- Last paragraph of page 5 the abbreviation FGM is used multiple times and full word is missing please add that. added
- Page 10, second paragraph "The review identified [118-137]." This sentence seems incomplete.completed the sentence
- Page 10, third paragraph 3rd line should be 2% to 86.6%. added
- Page 11, first paragraph the message is not very clear. Please elobrate further. Rephrased to make it clear.
- Figure 4 is incomprehensible in the PDF file please modify it. Size increased.

(continue on the next sheet)

Continued:

Discretionary revisions:

- Methods section can be more detailed to elucidate how the search was conducted.
  Methods are now reported in detail.
- Authors can relook at genetic counseling section although it is important but is not directly related to the recommendations and conclusion they are making. They may consider revising this part.
  Revisited the section and conclusion are modified on page 14.
  "Although limited evidence was found on genetic screening and counseling, it was reported that couples are generally receptive to such services. Therefore, comprehensive genetic counseling to all couples planning a pregnancy and genetic screening services to women is worthy particularly for those where the regional prevalence of genetic disorders are high. Providing preconception care that incorporates reproductive planning and genetic counseling can positively influence health in adolescents, young women and couples, and avert many negative MNCH outcomes over the next generation"

Referee's comments to the authors– this sheet <u>WILL</u> be seen by the author(s) and published with the article

Title	**Preconception care: promoting adolescent health and reproductive planning**
Author(s)	Sohni V Dean, Zohra S Lassi, Ayesha M Imam, Zulfiqar A Bhutta
Referee's name	Rehana Salam

When assessing the work, please consider the following points, where applicable:

1. Is the question posed by the authors new and well defined?
2. Are the methods appropriate and well described, and are sufficient details provided to replicate the work?
3. Are the data sound and well controlled?
4. Does the manuscript adhere to the relevant standards for reporting and data deposition?
5. Are the discussion and conclusions well balanced and adequately supported by the data?
6. Do the title and abstract accurately convey what has been found?
7. Is the writing acceptable?

Please make your report as constructive and detailed as possible in your comments so that authors have the opportunity to overcome any serious deficiencies that you find and please also divide your comments into the following categories:

- Major Compulsory Revisions (which the author must respond to before a decision on publication can be reached)

- Minor Essential Revisions (such as missing labels on figures, or the wrong use of a term, which the author can be trusted to correct)

- Discretionary Revisions (which are recommendations for improvement but which the author can choose to ignore)

Where possible please supply references to substantiate your comments.
When referring to the manuscript please provide specific page and paragraph citations where appropriate.

General comments:
A huge body of knowledge but not presented clearly.

Major compulsory revisions:
The title suggests that the authors will evaluate **<u>adolescent health and reproductive planning</u>**, while the paper doesn't sufficiently cover **<u>"adolescent health"</u>** as adolescent health in itself is a vast domain. Suggest changing the title to reflect what was done in the review.
We have now changed the title and restricted it to Reproductive planning.

The methodology paper and the methods section in this paper elucidate that the review focused on preconception care with a view to improvement in MNCH outcomes. Promoting adolescent health includes reducing dating violence and sexual coercion, encouraging cessation of FGM, and multi-faceted interventions that look at adolescents holistically (educational and vocational support, sex education, medical care, sports and arts, free STI testing, condom use... community service and personal development) to prevent adolescent pregnancy. The scope was not limited by the aim of the intervention, but rather by the outcomes reported and their link to preconception care. Further, it is emphasized that narrow programs focusing on sex education or abstinence have not been as effective as these broader programs that promote adolescent health and personal development.

We have now restricted it to Reproductive health only.

Introduction seems to be hastily written and lacks flow
The introduction has been amended to make it more concise and clear.

In introduction, 1st paragraph, "**For 1.2 billion adolescents…."** Needs to be updated with the recent data revised

Please define briefly "preconception care" in the background to give readers a clearer idea of what the review is about
We have suggested a reference to Paper 1 of the series where this has been explained and described in detail.

In methods section **"Standard criteria were applied to determine the quality of the evidence, and standard methods for meta-analyses of quantitative studies were used."** Please mention which criteria..
Revised and described the methodology in detail now.

In methods section **"The findings were presented at international meetings and shared with professionals in the relevant fields of maternal and child health, following which results were updated based on current searches (through end of 2012) and expert opinion."** What changes were made after the expert consultation? Were the changes significant enough to be mentioned in the paper??

The reference to those meeting has been provided. No change in the review was made but we revised the search and results where current evidence was found.

The methods section is not clear. It presents the paper as a systematic review and meta-analysis, however the results sections mentions findings from existing reviews… please describe the methodology more clearly

Described in detail.

The methods section should also clarify that the authors reviewed both the **interventions** and the **risks**

Clarified: "We systematically reviewed all literature published up to 2011 to identify studies describing the effectiveness of preconception (period before pregnancy and between pregnancy) interventions and risks for adolescent health and reproductive planning on maternal, newborn and child health outcomes"

Results section should not include parts of introduction/ background. Parts of results are already mentioned in the background. Please remove

Removed results from background.

In the results section, Suggest discussing the concordance and discordance with other reviews in the discussion section

Have moved discussion, including agreement or dissent with findings of previous reviews, to the conclusion

The results section of the abstract needs to be re-written as it clearly misses out on many interventions reviewed.

These are the only two interventions for which substantial evidence was found.

Minor essential revisions:

The paper starts with the heading **'Why adolescent health and reproductive planning are important from the preconception perspective'.** This looks more like a statement then a heading. Can be replaced with "**Background" OR "introduction"** since the title already gives an idea that it relates to preconception care.

Changed to Background

In introduction 1st paragraph, "**Adolescent girls and young women are particularly vulnerable, facing interpersonal violence, coerced intercourse, sexually-transmitted infections especially HIV, undernutrition or obesity and their health consequences; besides the social pressures that prevent them from completing their education and force them into early marriages and childbearing."** very long sentence, suggest clarifying and shortening

Shortened "Many adolescent girls and young women faces challenges such as interpersonal violence, coerced intercourse, sexually-transmitted infections especially HIV, undernutrition or obesity and their health consequences which makes them highly vulnerable. Social pressure on top of these, prevent them from completing their education and force them into early marriages and childbearing.

In Introduction, last paragraph, "**Millions of women and children, and future generations will benefit if we invest in Preconception care to improve adolescent health and encourage reproductive planning."** Looks like a concluding statement. Suggest removing it

Changed it a bit: instead of will, added can "Millions of women and children, and future generations can…"

In Introduction, last paragraph**, "This paper presents the findings of a systematic review that was undertaken to consolidate the evidence for risks and interventions relating to this area. It begins**….." needs to be re-written, not clear

Clarified: "This paper presents the findings of a systematic review that was undertaken to consolidate the evidence for risks and interventions relating adolescent health and reproductive planning during preconception period"

Can't see figure 4: added

In the results section, "reproductive planning after a pregnancy lost", 2nd paragraph **"The review identified ..". please complete the sentence**

Completed "The review identified 20 studies [118-137]."
